# Is juvenile dermatomyositis a different disease in children up to three years of age at onset than in children above three years at onset? A retrospective review of 23 years of a single center’s experience

**DOI:** 10.1186/1546-0096-10-34

**Published:** 2012-09-20

**Authors:** Anjali Patwardhan, Robert Rennebohm, Igor Dvorchik, Charles H Spencer

**Affiliations:** 1 University of Missouri Medical Center, Columbia, MO, USA; 2 Cleveland Clinic, Cleveland, OH, USA; 3 Nationwide Children’s Hospital Columbus, Columbia, MO, 4320, USA; 4 Department of Child Health, University of Missouri HSC, One Hospital Drive, Columbia, MO, 65212, USA

**Keywords:** Juvenile dermatomyositis, Young age at onset, Vasculopathy, Disease outcome

## Abstract

**Background:**

We tested the hypothesis that the course and outcome of juvenile dermatomyositis (JDM) in children seen at one center with the JDM disease onset at or below three years of age is different from that in the children with disease onset at greater than three years of age.

**Methods:**

Institutional Review Board approval was obtained to retrospectively review the charts of 78 patients from age 0–18 years with JDM seen in the pediatric rheumatology clinic at Nationwide Children’s Hospital in Columbus, Ohio over the past 23 years from January 1988. The diagnosis was made by the treating pediatric rheumatologist. Not all the patients met the Bohan and Peter criteria, as muscle biopsy and EMG were not always performed and we utilized a modified JDM criteria. The data regarding disease course and outcome were collected as of the last clinic follow-up or to July 1, 2010. We used the Wilcoxon Two-Sample test to compare numerical variables between two age groups, and used logistic regression to compare categorical variables between two age groups in SAS 9.1.3. Minitab-16 was used to calculate various mean, median, modes, standard deviations and range. For survival analysis, we used Kaplan-Meier method with log-rank test.

**Results:**

The mean age of onset in the two groups at Nationwide Children’s Hospital was 27 months and 91 months. The mean times between onset of symptoms to diagnosis in the younger and older age groups was 5.6 months and 4.5 months, respectively, not a statistically significant difference. The younger onset group had more females (p=0.05) and their disease onset occurred less frequently during the typical winter-spring seasons (p=0.031). The younger onset group was more likely to have a preceding fever (p=0.029) and family history of autoimmune diseases (p=0.012). The younger onset group was less likely to have heliotrope rash (p=0.04), Gottron’s sign (p=0.049), capillary loop abnormalities (p=0.010), or elevations in creatine kinase (CK, p=0.022), aspartate aminotransferase (AST, p=0.021) or aldolase (p=0.035). The younger onset group was treated less often with pulse methylprednisolone at diagnosis (p=0.043) and less often with hydroxychloroquine (p=0.035). There were no differences between the two groups regarding initial oral steroid dose (p=0.8017), number of patients who received methotrexate at diagnosis (p=0.709), and the number who ever received other immunosuppressants (p=0.323). The mean and maximum duration (mean duration 24.3 months vs. 35.2 months, maximum duration 51 vs. 124 months in younger and older onset group respectively) of methotrexate therapy, and the mean and maximum duration of oral steroid therapy (Mean duration 16.8 months vs. 33.3 months, maximum duration 50 vs. 151 months in younger and older onset group respectively), was shorter in the younger group. The younger onset patients were less likely to have active disease at 5 years (9% vs. 35.7%, p=0.015) and 10 years post-diagnosis (9% vs. 45.1%, p=0.011, Table 7). The younger patients were less likely to have osteonecrosis (p=0.023). Two disease-related deaths occurred in the younger group, none in the older group. The results of the survival analysis showed that the difference between the age groups was statistically significant (p < 0.012). The sex and race were not significant (p> 0.26 and p>0.95, respectively).

**Conclusions:**

There were significant differences between JDM patients with disease onset at or below age three years at our center, compared to their older counterparts. Younger patients in our cohort had fewer typical findings at diagnosis and a milder disease course without needing as long a duration of corticosteroids and immunosuppression. Patients with a younger onset had a higher mortality rate but mortalities were unusual and numbers small. The younger group had a similar complication rate compared to the older onset patients, except for osteonecrosis which was higher in the older onset group. These findings differ from the previous reports that a younger age of onset in JDM is often associated with a more severe disease, as results at our center suggest that children with younger onset JDM appear to be atypical but may do well compared to the older JDM patients.

## Background

Juvenile dermatomyositis (JDM) is an autoimmune disease of blood vessels in children and adolescents that primarily affects muscles and skin. The mean age of onset is 7 years of age and girls have a slight predominance [1]. The presenting features may include a disease pattern of an erythematosus rash on the cheeks, a red to violacious rash on the eyelids with edema, raised red papules on the extensor surfaces of finger joints, abnormal nailbed capillaries, and a red rash on the elbows, knees, and ankles. The hallmark feature is proximal muscle weakness that may be severe and debilitating [1,2].

The course of juvenile dermatomyositis has improved dramatically over the last 60 years. Prior to the 1950’s when daily corticosteroids started to be used consistently, 1/3 of JDM children recovered, 1/3 of JDM children were disabled, and 1/3 died [3]. Now after earlier diagnosis and aggressive treatment with corticosteroids and immunosuppressive drugs, JDM patients do much better with much less morbidity and mortality (<1% mortality) [4-12]. Prognostic factors are still lacking that might direct pediatric rheumatologists to the best treatment approach for each child as the experiences vary with different patient populations and research centers [9-12].

At Nationwide Children’s Hospital, the rheumatology service has often cared for young children with the onset of JDM at three years or less over the last 25 years. The disease course of JDM children we have seen varies considerably, making individual prognosis and treatment needs difficult to predict. There is little published literature comparing clinical presentation, disease course, and outcome of juvenile dermatomyositis in young children versus older children (e.g. disease onset below and above age 3 years). It was our hypothesis that this subset of JDM children may be different than the patients with an older onset of the disease at our institution in their clinical features, course, and treatment. In this study we therefore investigated age-specific disease characteristics and differences that may influence JDM disease course and treatment options using our cohort of JDM patients with a relatively large number of younger onset patients.

## Methods

Nationwide Childrens Hospital Institutional Review Board (IRB) approval was obtained for this study. We identified 85 patients with a medical record diagnosis (ICD-9 coding) of JDM at Nationwide Children’s Hospital from January 1988 to July 2010. After a preliminary review of electronic medical records and paper charts, 7 patients were excluded because they developed a rheumatic overlap syndrome or mixed connective tissue disease. We included 78 patients, all younger than 18 years of age at onset of disease. Nineteen were at or below 3 years of age and 59 were above 3 years of age at onset of symptoms of JDM. These charts were reviewed retrospectively to collect information for a period starting at their date of presentation to the last clinic follow-up, or to July 1, 2010. As a part of the rheumatology practice, the standard format of outpatient assessment clinical charts designed for JDM were used in clinics. After initial assessment/admission the patients are seen every 6–12 weeks in most cases depending on their individual needs. We collected information from the patient’s charts at various time points such as at the diagnosis, at 6 months post diagnosis, 1 year, 3 years, 5 years and then 10 years post-diagnosis. Demographic data was collected from medical record of all the JDM patients. Only 23 patients had race/ethnicity information in medical records; thus information on this variable, although important, could not be included. We examined the raw data from our retrospective chart review and found significant differences in the several disease aspects such as demographic and outcome parameters between those who had onset at three years or below compared to those above three years of age.

We used a modification of the diagnostic criteria proposed by Bohan and Peter [3]. The original criteria permit a definite diagnosis of dermatomyositis if there are characteristic cutaneous changes, and 3 of 4 other manifestations: symmetric proximal muscle weakness, elevated muscle enzymes (CK, AST, LDH, or aldolase), electromyographic evidence of myopathy and denervation, and characteristic muscle histopathology. Probable JDM is defined by the Bohan and Peter criteria as the presence of characteristic cutaneous changes and two of the four other manifestations.

In our modification of the Bohan and Peter criteria, we included typical abnormalities on muscle magnetic resonance imaging (MRI) as a 5th manifestation. When performing MRI on JDM patients at Nationwide Children’s Hospital in recent years, a standardized MRI protocol sequence was used. MRI is considered a highly sensitive tool for the diagnosis of muscle inflammation. Although it has low specificity; it provides information on muscle inflammation with almost same specificity as an EMG would. In a typical JDM MRI, we look for documented extent and intensity of the muscle abnormalities. The inflammation is usually symmetric and classically involves the proximal muscle groups in dermatomyositis. We look for high signal intensity in the active phase on STIR and fat-saturated gadolinium-enhanced T1-weighted images as a part of JDM protocol for MRI. In the chronic phase, we consider fatty atrophy of the musculature that is seen on T1-weighted images as suggestive of IIMD/JDM [13-15].

**Figure 1:**
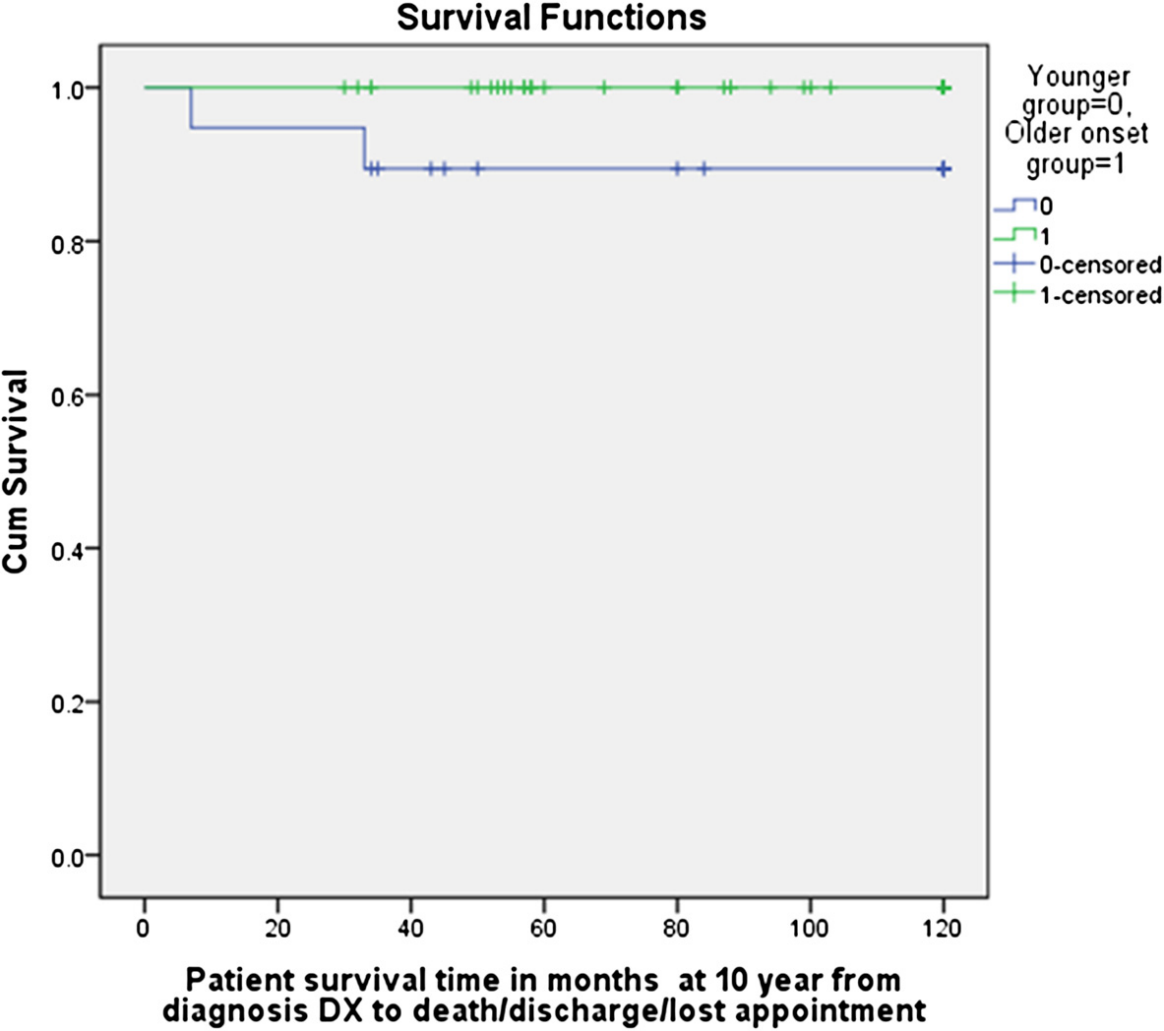
**Survival analysis: Kaplan-Meier method with log-rank test for comparison of survival curves.** The results of the survival analysis show, the difference the age group was statistically significant (p < 0.012). The Sex and race were not significant (p > 0.26 and p > 0.95, respectively). Censored = some of the subjects had not experienced the outcome of interest by the end of the follow-up period of ten years because they had dropped out, i.e., discharged, lost to followup or died before reaching the outcome of interest.

For our study purposes, definite JDM was defined as characteristic cutaneous changes plus 3 of 5 other manifestations, and probable JDM as characteristic cutaneous changes plus 2 of 5 other manifestations. Of our 78 patients, 14 from the younger onset group and 56 from the older onset group had definite dermatomyositis according to these modified criteria. Six patients (4 from the younger onset group and 2 from the older onset group) never developed significant rash before treatment, but were included in the study due to the muscle biopsy results very typical for JDM (Figure 1). Two patients included in this study were classified as probable JDM (one each from the younger and older onset groups).

**Table 1:** Differences in demographics and seasonal variations

**Demographics**	**3 years of age and below at onset n = 19**	**Above 3 years of age at onset n = 59**	**Total patients**	**p-value**
**Average age of onset in months**	27	90	78	
**Females: Males**	16/3 (5.3:1)	35/24 (1.4:1)	78	
**Onset Late Winter – Early Spring (January-May)**	11 (57%)	46 (78%)	78	0.031
**Onset Summer (June-September)**	5 (26%)	6 (10%)	78	0.042
**Onset Autumn (October-December)**	3 (15%)	7 (12%)	78	0.657

The evidence of active disease was assessed though clinical muscle weakness, active skin rash, abnormal muscle enzymes, neopterin concentrations, von Willebrand antigen level, and histological evidence with or without elevated inflammatory markers. No single physical exam muscle strength tool, such as the CMAS [16], was used in all cases in this study, due in part to the difficulty of accurately assessing strength in very young children and the lack of validity of these tools at that young age. For this reason, CMAS results are not reported in this study for patients seen after the CMAS was developed. The muscle strength testing in younger children had to rely more on observation of gross motor functioning than classic muscle testing or the CMAS, but the testing by these three experienced pediatric rheumatologists was felt to be accurate and reliable.

The following information was collected from both groups: Demographic details, season at onset, clinical presentation, lab results, disease course, treatments used, response to treatment, initial and final heights, complications, and outcome at 1, 5, and 10 years post-diagnosis. We recorded the time required to achieve the first remission and also time required to reach normal muscle strength for the first time after disease onset. We also compared the time between the onset of symptoms and diagnosis, patterns of remissions and relapses, and the effect of disease on growth of these patients.

As JDM frequently goes into remission within 2–3 years of treatment initiation [3,4,13,14], three years may be considered as one disease cycle. Different researchers have defined disease course differently [1]. For the purposes of this study, the course of the disease was classified as follows;

### Monocyclic course

The diseases course with a single episode of the disease with only one remission and no relapses.

#### Polycyclic

More than one remission and relapse in the diseases course of any duration.

### Chronic continuous

Continuously active disease course over 4 years without definite remissions.

### Relapse

Recurrence or increase in the clinical, lab or radiologic parameters of disease activity in a patient in whom such parameters had either improved or were reduced to reflect lower disease activity. Data collected for this was from physical examination of skin i.e. rash (Gottron’s, heliotrope/nailfold capillary abnormalities), muscle strength on clinical examination, MRI (JDM protocol), muscle enzymes) as applicable.

### Remission

Absence of clinical and lab/imaging parameters of disease activity for 6 months or more without any treatment. The data collected for this parameter was the same as for the relapse.

In the standardized charts for JDM patients, the patients were assessed for complications at the time of diagnosis and thereafter during each clinic visit. Three patients in this study, which included one from older age group and two from younger age group, had an ulcerative course of disease. Both the patients from younger age group died due to their disease (see Appendix A & B for the details).

## Statistics

We use Wilcoxon Two-Sample test to compare numerical variables between two age groups, and use logistic regression to compare categorical variables between two age groups in SAS 9.1.3. Minitab-16 was used to calculate various mean, median, modes, standard deviations and range. For survival analysis, we used Kaplan-Meier method with log-rank test.

## Results

The mean age of onset in the two groups of JDM patients seen at Nationwide Children’s Hospital was 27 months and 91 months. The mean intervals between onset of symptoms to diagnosis in younger and older age groups were 5.6 months and 4.5 months respectively. The difference was not statistically significant. The younger onset group had more females than males (p=0.05) and their disease onset occurred less frequently during the most common JDM onset peak of the late winter-spring months (January to May, p = 0.031) [15-17] (Table 1).

**Table 2:** JDM features at diagnosis

**Disease features**	**3 years of age and below at onset n = 19**	**Above 3 years of age at onset n = 59**	**Total patients**	**p-value**
**Preceding fever**	14 (73%)	21 (35%)	78	0.029
Other systemic symptoms*	5 (26%)	14 (23%)	78	0.82
**No heliotrope Rash**	7 (36%)	8 (13%)	78	0.04
**No Gottron’s papules**	9 (47%)	4 (6.8%)	78	0.049
**No rash at all**	4 (21%)	2 (3%)	78	0.039
**No Capillary Loop abnormality**	12 (63%)	5 (8%)	78	0.010
**Swallowing difficulties**	3 (15%)	7 (11%)	78	0.66
**Raynaud’s phenomenon**	1 (5%)	5 (8.4%)	78	0.048
**Family history of Autoimmune disorders**	15 (80%)	26 (44%)	78	0.012

* Recurrent troublesome abdominal pain, bloody diarrhea, shortness of breath, tachycardia, syncope, swallowing difficulties, nasal regurgitation, central nervous system symptoms.

{**Swallowing difficulties **= 3/19 in younger group, 5/59 in older age group.

**Shortness of breath/ tachycardia/ syncope** = 2/19 in younger group, 10/59 in older age group.

**Abdominal pain/bloody diarrhea/nasal regurgitation **= 4/19 in younger group, 10/59 in older age group.

**Symptoms involving central nervous system** = 1/19 in younger group, 2/59 in older age group.

**Generally patients had more than one type of systemic symptom**.}

The younger onset group was more likely to have a preceding fever (p=0.029) and family history of autoimmune diseases (p=0.012). Patients in the younger onset group less often had heliotrope rash (p=0.04), Gottron’s sign (p=0.049), or nailfold capillary loop abnormalities (p=0.010), or any JDM rash at all (p=0.049) (Table 2). They also had a lower frequency of muscle enzyme elevation such as creatine kinase (CK, p=0.032), aspartate aminotransferase (AST, p=0.021), aldolase (p=0.035), and lactate dehydrogenase (LDH, p=.042) (Table 3), than the patients in the older age group.

**Table 3:** Normal muscle enzymes, ESR, WBC and hemoglobin at diagnosis (diagnosis)

**Labs at diagnosis**	**3 Years or below age group n = 19**	**Above 3 years of age group n = 59**	**Total patients**	**p-value**
**Normal AST**	14 (73.6%)	23 (38.9%)	78	0.021
**Normal Aldolase**	14 (73.6%)	20 (33.8%)	78	0.035
**Normal LDH**	15 (78.90% )	25 (42.30%)	78	0.042
**Normal CK**	14 (73.60%)	25 (42.3%)	78	0.032
**Elevated ESR**	12 (63% )	26 (44%)	78	0.152
**Elevated WBC**	None (0)	4 (7%)	78	-
**Low Hb (<11gm/dl)**	9 (47%)	21 (35%)	78	0.361

**Table 4:** Management at diagnosis

**Management at diagnosis (Within 4 weeks of the diagnosis)**	**3 Years of age and below at onset n = 19**	**Above 3 Years of age at onset n = 59**	**Total patients**	**p-value**
**Received admission at diagnosis**	6 (31%)	36 (61%)	78	0.043
**Initial dose of oral prednisone**	Dose in mg /kg/day	Dose in mg/kg/day	78	-
	n = 16	n = 54		
	Mean = 1.4	Mean = 1.423		
	St Dev = 0.504	St Dev = 0.5237		
	Median = 1.450	Median = 1.500		
	Range = 0.75 to 2	Range = 0.27 to 2		
	Mode = 2	Mode = 2		
**Received pulse steroids at the diagnosis**	6 (31%)	35 (59%)	78	0.043
**Received treatment with methotrexate at the diagnosis**	12 (63%)	40 (68%)	78	0.709

The younger onset group was treated less often with pulse methylprednisolone (p=0.043) and hydroxychloroquine (p=0.035). These younger children were less likely to be admitted to the hospital (p=0.043) at the time of diagnosis. There were no differences between the two groups in the initial oral steroid dose in mg/kg/day (1.4 mg/kg/day in both the age groups, p=0.90), the proportion of patients who received methotrexate at diagnosis (p=0.70), and the proportion of patients of each group who ever received other immunosuppressive medications during their disease course (p=0.32) (Tables 4 and 5). No differences in the frequency of initial use of steroids and use of methotrexate at diagnosis were found between the two age groups. The mean and maximum duration of methotrexate therapy (mean duration 24.3 months vs. 35.2 months, maximum duration 51 vs. 124 months in younger and older onset group respectively), and the mean and maximum duration of oral steroid therapy (mean duration 16.8 months vs. 33.3 months, maximum duration 50 vs. 151 months in the younger and older onset group respectively), was shorter in the younger group.

**Table 5:** Treatment received during the course of the disease

**Treatment received**	**3 Years of age and below at onset n = 19**	**Above 3 years of age at onset n = 59**	**Total patients**	**p-value**
**Plaquenil**	1 (5%)	17 (29%)	78	0.035
♦ **Immunosuppressant**	3 (16%)	16 (27%)	78	0.323
**IVIG**	3 (16%)	17 (29%)	78	0.27
**Rituximab**	0 (none)	3 (5%)	78	-
**• Duration in months of methotrexate therapy**	n=13	n = 48	78	-
	Mean = 24.31	Mean = 35.17		
	St Dev = 15.22	St Dev = 27.20		
	Median = 23.00	Median = 26.00		
	Range = 1 to 51	Range = 4 to 124		
	Mode = 12	Mode = 17, 25		
**• Duration in months of oral prednisolone/prednisone therapy**	n = 18	n = 55	78	-
	Mean = 16.89	Mean = 33.33		
	St Dev = 13.62	St Dev = 27.62		
	Median = 19	Median = 23		
	Range = 1 to 50	Range = 4.5-151		
	Mode = 19	Mode = 14, 15, 21, 36		
♦ **Non-methotrexate immunosuppressant: anti-tumor necrosis factor biologics, mycophenolate mofetil, cyclosporine and cyclophosphamide**				

In the younger group: 12/19 patients were started on MTX at the diagnosis but eventually 13/19 received methotrexate during their disease course. While in patients with older onset group: 40/59 patients were started on MTX at diagnosis but eventually 48/59 patients received methotrexate during their disease course.

Examination of the disease outcome at 1 year, 5 years, and 10 years revealed differences between the two onset groups of JDM patients (Table 6). Consistent with their onset characteristics, at one year post-diagnosis, younger onset patients were significantly less likely to have elevated muscle enzymes than older onset patients (0% vs. 15%, p=0.018). There was also a trend for a lower persistence of muscle weakness (26% vs. 37%, p=0.461) and rash (32% vs. 54%, p=0.126) in the younger onset group compared to the older group, but neither comparison reached statistical significance. The younger onset patients were less likely to have active disease at 5 years (9% vs. 35.7%, p=0.015) and 10 years post diagnosis (9% vs. 45.1%, p=0.011) (Table 7). Younger age group patients were more likely to experience a monocyclic course (84.9% vs. 33.8%, p=0.027) and less likely to experience a polycyclic (p=0.019) or chronic continuous course (p=0.03) (Table 7).

**Table 6:** Course of JDM at 1 year, 5 year, and 10 years from the date of diagnosis

**Time / Disease course**	**3 Years of age and below at onset n = 19**	**Above 3 years of age at onset n = 59**	**Total patients**	**p-value**
**1 Year**				
**Persistent muscle weakness**	5/19 (26%)	22/59 (37%)	78	0.4612
**Persistent rash**	6/19 (31%)	32/59 (54%)	78	0.1261
**Elevated muscle enzymes**	0/19 (none)	15/59 (25%)	78	0.018
**5 Years**			78	
**Active disease**	1 (9%) (8/19 patients were discharged/lost followup)	15 (35.7%) (17/59 patients were discharged/lost followup)	53	0.015
**10 years**				
**Active disease**	1 (9%) (8/19 patients were discharged/lost followup)	14 (45.1%) (28/59 patients were discharged/lost followup)	42	0.011

**Table 7:** Disease course response patterns

**Disease course**	**Frequency in 3 years of age and below at onset n = 19**	**Frequency in above 3 years of age at onset n = 59**	**p-value**
**Monocyclic Disease**	16 (84.2%)	20 (33.8%)	0.027
**Polycyclic**	1 (5.2%)	21 (35.5%)	0.019
**Chronic continuous**	2 (10.5%)	18 (30%)	0.03
**Time in months from diagnosis to first remission**	n = 13	n = 37	-
	Mean = 23.62	Mean = 26.84	
	St Dev = 13.72	St Dev = 14.91	
	Median = 21	Median = 24	
	Range = 6 to 51	Range = 7 to 78	
	Mode = 6, 19	Mode = 23, 27	
**Duration in months of disease follow up**	n = 19	n = 59	-
	Mean = 78.4	Mean = 95.10	
	St Dev = 61.0	St Dev = 62.12	
	Median = 56.0	Median = 81	
	Range = 6 to 202	Range = 6 to 211	
	Mode = 36, 85	Mode = 21, 24, 26	
**Time in months to reach near normal muscle strength first time**	n = 9	n = 41	-
	Mean = 10.33	Mean = 8.29	
	St Dev = 10.37	St Dev = 10.53	
	Median = 6	Median = 6	
	Range = 5 to 37	Range = 1 to 62	
	Mode = 6	Mode = 6	

**Table 8:** Histochemical findings of biopsy tissue of patients who did not have classic JDM rash (heliotrope, nail bed capillary changes and Gorton papules)

**Patient number**	**Deposition of MAC (membrane attack complex)**	**Perivascular and perifascicular inflammatory Infiltration (The predominantly B cells & CD4+ T cells)**	**Perifascicular necrosis/ atrophy**	**Vasculopathic changes in capillaries/reduced capillary number**	**Myocyte necrosis and regeneration**	**Special comments**
DM1	+	+	+	+	+	
DM2	_	+	+	+	+	Received oral steroids for a month before biopsy
DM3	+	+	+	+	+	
DM4	+	+	+	+	+	
DM5	+	+	+	+	+	
DM6	-	+	+	+	+	Received two IV pulse steroids before biopsy

Complications were evaluated, including serious infections (patients requiring IV antibiotics and/or hospital admission for treatment of infection), calcinosis, muscle atrophy, lipodystrophy, osteonecrosis, growth retardation, and death. The younger patients were less likely to have osteonecrosis (5% vs. 17%, p=0.023). Though the differences in most complication rates did not reach statistical significance, there were trends. The rates of serious infections (36% vs. 29%), muscle atrophy (21% vs.15%) and lipodystrophy (16% vs.13%) were slightly higher in the younger onset group of children, but the rate of calcinosis (15% vs. 22%) was slightly lower. The data on weight percentiles was not analyzed, because the relative effects of disease activity and steroid treatment on weight changes could not be determined and also variation on the growth velocity in the two groups might have been a major confounder.

Three of the patients had an ulcerative JDM course. The patient from the older group had chronic continuous course while both the patients from younger age group died due to their disease (see Appendix A & B for short clinical histories). Time in months from diagnosis to reaching near normal muscle strength for the first time was longer in younger onset age group (10.33 vs. 8.29) as compared to older age group but inability to test muscle strength due to poor cooperation may be a huge confounder in younger age of onset group.

For survival analysis, we used Kaplan-Meier method with log-rank test for comparison of survival curves (Figure 1). The results of the survival analysis show that the differences between the age groups were statistically significant (p < 0.012). The sex and race were not significant (p > 0.26 and p > 0.95, respectively).

In contrast to their milder overall course, disease-related deaths were more frequent in the younger onset age group (2) than in the older onset group (0) (p < 0.02). One of the 2 patients in the younger group who died had severe ulcerative disease involving the skin and intestinal tract (see Appendix A and B).

One potential bias is the use of immunosuppressive medications. The immunosuppressive drugs used to treat JDM have evolved over past 23 years. In our institution, the immunosuppressives drugs were used over following approximate time lines: methotrexate by 1980, cyclosporine by 1990, mycophenolate mofetil (MMF) by 2000, TNF-α inhibitors and B-Cell depletion from 2000. We believe that this time course of treatment is typical of pediatric rheumatology centers with access to these drugs and ability of patients to afford these drugs.

## Discussion

The goal of this study was to compare the onset characteristics and course of dermatomyositis in very young children (≤3 years old) and in older children. We recognized that establishing a diagnosis of JDM can be problematic using the 1975 criteria of Bohan and Peter [3], due to difficulty in assessing muscle strength in younger children, and a reluctance to perform invasive procedures for diagnosis in both younger and older children when the clinical pattern looks very typical for JDM. In clinical practice, many pediatric rheumatologists currently use the MRI, particularly of the thigh and hip girdle muscles, as a noninvasive method to identify proximal muscle inflammation. Fat-suppressed T2-weighted sequences or STIR (short tau inversion recovery) sequences are sensitive for demonstration of muscle edema, which typically occurs in a patchy but relatively symmetric distribution in JDM [13-15]. The addition of typical MRI abnormalities was ranked highly in a recent consensus conference to develop revised criteria for JDM. Consistent with newer trends in pediatric rheumatology practice and the likely future inclusion of MRI in formal diagnostic criteria for JDM, we added typical MRI abnormalities to the diagnostic criteria for this study. The data was collected retrospectively from the standardized forms/clinical charts designed for JDM patients and used in rheumatology clinics at Nationwide Childrens Hospital.

Age characteristics of our JDM population were similar to those already described in the literature. In our study, the ratio of female to male patients overall was 1.9:1, which compares well with pooled data on JDM patients from diverse geographical areas especially, around North America, showing a gender ratio of 1.5:1 to 2.3:1 [17-20]. In contrast, the female to male ratio in the United Kingdom and Ireland has been reported as high as 5:1 [21]. In our study, the female to male ratio of patients with onset ≤ 3 years was more skewed towards females (3.5:1) as compared to the older onset group (1.4:1). The age-dependent variation in the sex ratio has been widely reported in published literature [6-8,17-21]. In our study population the younger onset group had no seasonal clustering of disease onset. Sixty percent of these patients had fever preceding the onset of JDM. In contrast, the older onset group of JDM patients in our study population did have a seasonal clustering of the onset of disease during the late winter and spring, and only 38.9% had preceding fever. These findings may suggest the possibility of different etiological agents or environmental factors playing a significant role in disease pathogenesis in the two age groups, perhaps agents and factors that have been discussed in previous studies or ones not yet discovered [22-25].

**Figure 2:**
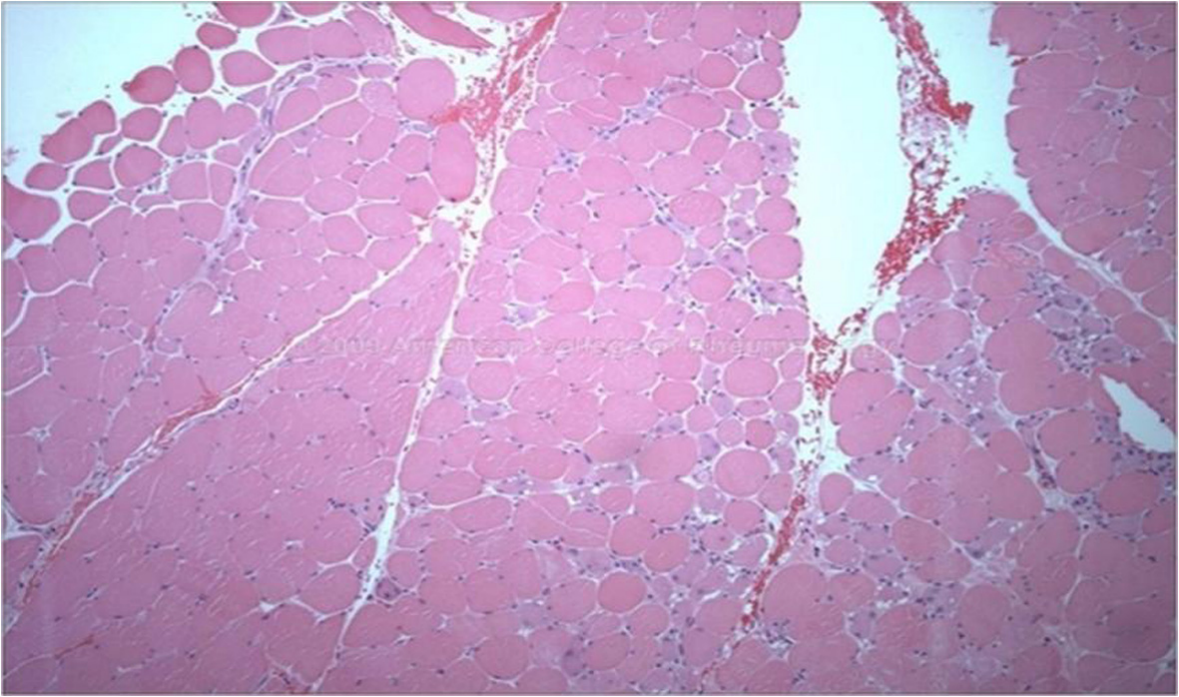
Histological features of muscle biopsy in JDM.

A significantly higher proportion of patients in the younger onset group of children presented without typical JDM rash or nailfold capillary loop abnormalities (Table 8). It is well known that onset of rash and myositis can be discordant in JDM [26,27]. In patients presenting without typical cutaneous manifestations of JDM, muscle biopsy was performed in 6 patients to rule out other muscle diseases and confirm a diagnosis of JDM (Table 8 and Figure 2). Another difference was that the younger onset group had less frequent muscle enzyme elevation at diagnosis. The diagnosis was not significantly delayed in the younger onset group compared to the older, though there was a trend for the younger group to have a longer time period from onset of the symptoms to diagnosis (5.7, 4.5 months).

Others have speculated that differences in disease trigger or host responsiveness to a trigger, genetic influences, or host maturity may affect disease course and outcome in JDM [28,29]. The differences we found between the younger and older onset groups may be reflective of such factors, but further speculation based on our results is not warranted.

A significantly lower proportion of patients from the younger onset group were admitted to the hospital and given intravenous pulse steroids at diagnosis, suggesting that younger patients may not have appeared to have very severe disease at presentation. It could also be that younger patients took a little more time to diagnose as most were evaluated only on an outpatient basis and over a longer time period.

In our study population, the initial dose of oral prednisone (mg/kg/day) and the proportion of patients treated with methotrexate at diagnosis were the same in the younger and older onset groups. This similarity may be a reflection of the standard of practice in this rheumatology division, or it may suggest a relative similarity in disease severity at onset in these groups of patients, despite the difference in hospitalization rate. The treatment was uniform within our group as any pediatric rheumatology center as a great majority of the patients were seen by two pediatric rheumatologists working closely together.

We found that patients in the younger onset group were more likely to have a monocyclic course and less likely to have active disease at 5 and 10 years from diagnosis. In unpublished data from a recent study from UK, there was no evidence that patients with younger age of onset had a worse disease course and outcome [30]. Both the more recent UK report and our findings contradict a previous report suggesting that a younger age of onset in JDM is associated with greater severity of disease and poorer outcome [31].

A significantly lower proportion of patients in the younger onset group of children received hydroxychloroquine as compared to the older onset group of children. This result again might be influenced by institution-based practices, and/or by the fact that younger children have difficulty taking a medication that is readily available only in pill form. Another explanation could be reluctance to prescribe hydroxychloroquine to younger patients due to greater difficulty in identifying and monitoring retinal toxicity.

The relative number of patients who were treated with other immunosuppressive medications in general (IVIG, TNF inhibitors, MMF, CSA, cyclophosphamide or azathioprine) was the same in the two groups. Rituximab was used in only two patients, and cyclophosphamide in only one patient, all in the older onset group of children. The reason for the use of these aggressive treatments in only the older patients could have been more resistant disease in this group, reluctance to use these medications in younger children, or skewing based on the presence of many fewer children in the younger group.

We looked at the means, medians, and longest durations of steroids and methotrexate courses in the two groups. There was a significant difference in the length of methotrexate and of steroid therapy between two groups. Patients from the older onset group of children received longer courses of both the drugs. This difference is consistent with the observation that the older onset group had a more protracted disease course.

There were trends towards an increased incidence of several complications in the younger onset group, and towards more calcinosis in the older onset group. The only difference in complication rate that reached statistical significance was osteonecrosis, which was higher in older onset patients. These patients received a higher average cumulative steroid dose, possibly increasing their risk for osteonecrosis. Their more protracted courses with more remissions and relapses, and presumably longer duration of active inflammation, also may have increased their risk for calcinosis. The younger group had a higher rate of mortality perhaps due to their higher vulnerability or higher number of ulcerative JDM in the younger age group (two in the younger and one in the older age group) but the numbers are too small to draw any conclusions. The survival analysis was performed to reduce the likelihood of bias that subjects have been censored (some of the subjects had not experienced the outcome of interest by the end of the followup period of ten years because they had dropped out, i.e. discharged, lost to followup, or died before reaching the outcome of interest). The younger group had less chance of this bias and had better followup.

The JDM disease with onset at three years or below was different than that of older children at our center. These may be ethnic factors or other epidemiological influences that may be unique to our area. In spite of two deaths, it would appear that the younger onset group did better overall in multiple parameters. The outcome was different for patients with ulcerative disease in the two groups in our study. The incidence of ulcerative disease was higher in younger onset population, as was the mortality in the patients with ulcerative disease compared to older onset JDM patients. Previously, different from our results, researchers have reported conflicting results. There has been no difference or worse disease in the younger group when the age of onset cutoff of 5 years was used between the two groups for comparison [23,24]. They also reported more use of cyclophosphamide and anti TNF-α in the younger onset JDM patients compared to older onset group of patients, which may suggest higher incidence of aggressive disease. They did not report explicitly on the ulcerative disease or mortality in their population.

A limitation of our study is that it is a retrospective study with variability in the clinical practice of the rheumatologists. Diagnostic approaches and treatment programs varied and may have had effects on disease response and course. Rheumatologists in the group all evaluated each patient by history, physical exam, and muscle enzymes but varied in the evaluation of the JDM patients by EMG, muscle biopsy, and MRI. Another limitation was that this is a single center report with 19 JDM patients in the 3 years or younger onset group. False positives are possible in a small research population. This is a statistical drawback but in the rare disease such as JDM, 19 patients with a disease onset at 3 years or below is unusual and worth studying.

## Conclusions

There were significant differences between JDM patients at our center with disease onset at or below age three years compared to their older counterparts. Younger patients in our cohort had fewer typical findings at diagnosis. They were more likely to experience a monocyclic course, a shorter and milder total disease course, and a shorter maximum duration of oral steroid and methotrexate therapy. Younger patients had predominantly monocyclic disease, and a lower proportion had active disease at 5 and 10 years. In spite of having a milder disease course overall, patients with younger onset had a higher mortality rate and a similar complication rate compared to the older onset patients, except for a significantly higher frequency of osteonecrosis in the older onset group. These differences in the mortality rate deserve further study with a larger sample size. We should continue to follow these younger children carefully and watch for ulcerative disease from the time of their diagnosis. Despite this mortality concern, our overall findings do differ from the previous report that a younger age of onset in JDM is usually associated with a more severe disease. These younger age children are atypical at our center, but do better than their older counterparts.

## Appendix A

First fatal case of JDMShe was a 3-year-old African American female who presented with arthritis and moderate muscle weakness. The significant positives and negatives in her physical examination and investigations were: positive nail bed capillary changes but no gottron or heliotrope, and a history of preceding fever. Her aldolase and creatine kinase (CK) was moderately elevated but AST and LDH were normal. Her ESR was 43, CBC was normal except mild normocytic normochromic anemia, her ENA’s were negative, and the anti-PSM/SLE test was indeterminate. Her myositis specific antibody panel was negative; an ANA titer was 1:1280 (speckled). A blind quadriceps biopsy showed patchy chronic inflammatory infiltrates and necrosis in perivascular perifascicular- perimysium regions with myofibril regeneration at various stages. There was reduced number of small vessels/capillaries with vasculopathic changes. She had histology-proven lipodystrophy at both prepatellar areas in the lower limbs. The fiber type and proportion was normal. There were no MAC-positive endomysial capillaries seen by immunoflourescence. There was minimal perimysial fibrosis and muscle atrophy also seen. There was no accumulation of glycogen, no abnormal inclusions, no vacuoles, and no access lipid content found in the myofibrils. The muscle biopsy was reported as consistent with JDM.During her 2.9 year-long disease course, she was treated with pulse steroids, oral steroids, methotrexate, azathioprine and IVIG. Compliance was always an issue with her. She did not show any clinical evidence of internal organ involvement. She had a difficult and resistant to treatment disease course. In her last few months she missed several appointments.One morning she was brought dead in emergency room (ER). The autopsy findings confirmed severe skeletal muscle inflammation with typical JDM histopathology. She also had chronic inflammatory changes in proximal skeletal muscle part of esophagus and acute-on-chronic gastritis (H. Pylori negative) with lymphoid hyperplasia of the terminal ilium. Histopathologically, her CNS was normal, but her heart was enlarged and had a patchy mixed inflammatory infiltrate in the myocardium with patchy myocardial necrosis. The infiltrate consisted of lymphocytes, histiocytes, and neutrophils. The areas of inflammation also showed contraction band necrosis. There was myocyte injury and necrosis surrounded by inflammatory cells in the myocardium. The pericardium was thickened and showed chronic inflammation and a moderate amount of pericardial effusion. The lungs showed chronic inflammatory infiltrates (mainly lymphocytes), mostly in the bronchi, bronchioles and interstitial tissue. A patch of consolidation is seen in right upper lobe. Her cause of death was ascertained as fulminating JDM disease activity and inflammation.

## Appendix B

Second fatal case of JDMShe was a caucasian female with an age of onset of JDM of 18 months. She started with an unusual presentation of having recurrent ulcerated lessons on the skin and tongue. She lost a part of the tip of the tongue in the process of healing. The histology of the skin ulcers showed no lupus band but a lymphocytic infiltrate and classic vasculopathic changes in the microvasculature. There was prominent angiocentric infiltrate composed of activated-appearing lymphocytes and histiocytes associated with endothelial swelling.She had no muscle weakness at the time. Her skin findings included a heliotrope rash which was photosensitive in nature. She did not have a Gottron’s rash or remarkable nail bed capillary changes. After extensive negative investigations for infections, her PCP imperially treated her with repeated courses of oral steroids. She relapsed when steroids were discontinued. After 6 months of initial symptoms, she sub-acutely developed moderate muscle weakness, difficulty in speaking and swallowing, and some loss of balance. She was referred to rheumatology.At that time the significant positives and negatives from her physical examination and investigations were: negative ANA, normal ESR, normal CK & aldolase, normal Von Willibrand antigen, normal GGT and ALT but moderately elevated AST and LDH. A Factor VIII antigen and von Willibrand antigen were elevated supporting JDM. Myositis-specific antibodies were negative. An MRI of the pelvic girdle on the JDM protocol showed substantial calcification in the muscles along with increased T2 signal in quadratus labiorum, erector spinae, iliopsoas, iliacus and inferior psoas suggesting ongoing, uncontrolled muscle inflammation. Extensive calcification was also found in costovertebral junctions, paraspinal muscles and anterior longitudinal ligaments. The biopsy of the paraspinal muscles was non-contributory as it was reported as extensive muscle degeneration and atrophy involving the majority of the histology section with a minimal perivascular inflammatory infiltrate.She was treated with pulse steroids, oral steroids, IVIG, and methotrexate for her illness. She had a difficult disease course, which only lasted for seven months. A bowel perforation due to vasculopathy and ischemic necrosis of her small intestine occurred and she developed a subdiaphragmatic abscess. She was hospitalized and recovered from this episode of illness and was discharged home. A few weeks later she was found dead in the bed. The autopsy report included: Her diaphragm showed chronic inflammation and was adherent to underlying liver. Several intra-abdominal adhesions between bowel loops and bowel wall were present. A healing subdiaphragmatic abscess, evidence of previous bowel resection, sub-capsular renal acute inflammatory process and significant cardiomegaly was also noted. The cause of death reported was also acute fulminating JDM disease and inflammation.

## Abbreviations

JDM: Juvenile dermatomyositis; AVN: Avascular necrosis; AST: Aspartate aminotransferase; LDH: Lactate dehydrogenase; CK: Creatine kinase; EMG: Electromyography; MRI: Magnetic resonance imaging; TNF: Tumor necrosis factor; MMF: Mycophenolate mofetil; MAC: Membrane attack complex; St Dev: Standard deviation.

## Competing interests

None to declare.

## Authors' contributions

Dr. Patwardhan and Dr. Spencer conceived of this study. Dr. Patwardhan did the chart reviews and data analysis. Dr. Rennebohm and Dr Spencer were instrumental in evaluation of the data and results. Dr. Patwardhan wrote the manuscript and the two senior authors helped in revisions and suggestions. Dr. Dvorchik performed the statistical analysis. All authors read and approved the final manuscript.
